# HSF1 is required for induction of mitochondrial chaperones during the mitochondrial unfolded protein response

**DOI:** 10.1002/2211-5463.12863

**Published:** 2020-05-15

**Authors:** Arpit Katiyar, Mitsuaki Fujimoto, Ke Tan, Ai Kurashima, Pratibha Srivastava, Mariko Okada, Ryosuke Takii, Akira Nakai

**Affiliations:** ^1^ Department of Biochemistry and Molecular Biology Yamaguchi University School of Medicine Ube Japan; ^2^ Present address: Key Laboratory of Animal Physiology, Biochemistry and Molecular Biology of Hebei Province College of Life Sciences Hebei Normal University Shijiazhuang Hebei 050024 China

**Keywords:** heat shock protein, HSF1, mitochondria, proteostasis, proteotoxic stress, SSBP1

## Abstract

The mitochondrial unfolded protein response (UPR^mt^) is characterized by the transcriptional induction of mitochondrial chaperone and protease genes in response to impaired mitochondrial proteostasis and is regulated by ATF5 and CHOP in mammalian cells. However, the detailed mechanisms underlying the UPR^mt^ are currently unclear. Here, we show that HSF1 is required for activation of mitochondrial chaperone genes, including HSP60, HSP10, and mtHSP70, in mouse embryonic fibroblasts during inhibition of matrix chaperone TRAP1, protease Lon, or electron transfer complex 1 activity. HSF1 bound constitutively to mitochondrial chaperone gene promoters, and we observed that its occupancy was remarkably enhanced at different levels during the UPR^mt^. Furthermore, HSF1 supported the maintenance of mitochondrial function under the same conditions. These results demonstrate that HSF1 is required for induction of mitochondrial chaperones during the UPR^mt^, and thus, it may be one of the guardians of mitochondrial function under conditions of impaired mitochondrial proteostasis.

## Graphical abstract

The mitochondrial unfolded protein response (UPR^mt^) is characterized by the transcriptional induction of mitochondrial chaperone and protease genes in response to impaired mitochondrial proteostasis. Here, we show that heat shock transcription factor (HSF1) is required for activation of mitochondrial chaperone genes and supports the maintenance of mitochondrial function in mouse cells during the UPR^mt^.

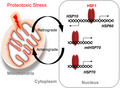

**Abbreviations**
ATF: activating transcription factor
C/EBP: CAAT enhancer‐binding protein
CHOP: C/EBP homology protein
HSF1: heat shock transcription factor 1
HSP: heat shock protein
HSR: heat shock response
SSBP1: single‐stranded DNA binding protein 1
UPR: unfolded protein response

Protein homeostasis or proteostasis within a cell is adjusted mainly at the levels of protein synthesis, folding, and degradation, and its maintenance is essential for cellular functions. Environmental and metabolic stresses constantly induce protein misfolding and challenge proteostasis capacity. To cope with these proteotoxic stresses, cells are equipped with adaptive mechanisms accompanied by changes in gene expression [1]. Among these, the heat shock response (HSR) is an evolutionarily conserved mechanism that is characterized by the induction of a set of heat shock proteins (HSPs) or chaperones, including HSP110, HSP90, HSP70, HSP40, and HSP27, which assist with protein folding, and some non‐HSP proteins involved in protein degradation [2]. The HSR is regulated mainly at the transcriptional level by heat shock transcription factor 1 (HSF1) in mammalian cells, and it maintains proteostasis capacity in both the nucleus and cytoplasm [3].

In contrast, analogous adaptive responses against protein misfolding in the endoplasmic reticulum (ER) and mitochondria are called as unfolded protein response in the ER (UPR^ER^) and mitochondrial UPR (UPR^mt^), respectively [4, 5]. The latter response is characterized by the induction of mitochondrial chaperones and proteases, which localize and act in the mitochondria in response to the accumulation of misfolded proteins or an imbalance in mitochondrial and nuclear‐encoded proteins in the mitochondria [6, 7, 8]. This response is regulated by the basic leucine zipper (bZIP) transcription factor ATFS‐1 in *C. elegans* [9]. ATFS‐1, which is localized to the mitochondrial matrix in normal conditions, accumulates in the nucleus and activates the UPR^mt^ genes in response to mitochondrial proteotoxic stress. In addition, several factors including a mitochondrial transporter, transcription factors, and histone‐modifying enzymes are also involved in the UPR^mt^ [10, 11]. In particular, histone demethylases JMJD‐3.1 and JMJD‐1.2 are necessary, and their overexpression is sufficient for the UPR^mt^ [12]. In mammals, the bZIP transcription factor ATF5 is regulated similarly to ATFS‐1 and activates the UPR^mt^ genes during accumulation of truncated ornithine transcarbamylase (ΔOTC) in the mitochondria [13]. Another bZIP transcription factor CHOP in complex with C/EBP also activates the UPR^mt^ genes, and its expression is induced via activation of JUN, which is mediated by c‐Jun N‐terminal kinase 2 during accumulation of ΔOTC [7, 14].

At first, synthesis of a mammalian homolog of the bacterial GroEL protein was found to be elevated during heat shock and was referred to as HSP58 (thereafter HSP60), whereas that of a mitochondrial member of HSP70 family was increased in cells deprived of glucose and was referred to as glucose regulated protein GRP75 (also known as mtHSP70) [15]. Mammalian HSP60 and HSP10 genes are linked head‐to‐head and share a bidirectional promoter, which is activated during heat shock [16, 17]. However, HSF1 was not thought to be involved in the upregulation of HSP60 and HSP10 during the UPR^mt^, because HSP70 was not upregulated simultaneously [6, 7, 16]. Recently, it was suggested that HSF1 in complex with a coactivator, mitochondrial single‐stranded DNA binding protein 1 (SSBP1), regulates the expression of mitochondrial chaperones, including HSP60, HSP10, and mtHSP70, during heat shock [18]. Of note, not only HSF1 but also mitochondrial SSBP1 accumulates in the nucleus and binds to the promoters of these genes on heat shock conditions [18]. Therefore, it should be determined whether HSF1 and SSBP1 play an indispensable role in the UPR^mt^. In this study, we showed that HSF1 is required for expression of nuclear‐encoded mitochondrial chaperones, HSP60, HSP10, and mtHSP70, but not for that of Lon protease, in response to impaired mitochondrial proteostasis, whereas SSBP1 is partially required for the induction. Furthermore, HSF1 promoted the maintenance of mitochondrial function during the UPR^mt^.

## Materials and methods

### Cell cultures and treatments

Immortalized wild‐type (clone #10) and HSF1‐null (clone #4) mouse embryonic fibroblasts (MEF) [19], HeLa (ATCC CCL‐2) cells, and HEK293 (ATCC CRL‐1573) cells were maintained at 37 °C in 5% CO_2_ in Dulbecco's modified Eagle's medium containing 10% fetal bovine serum (Sigma‐Aldrich St. Louis, MO, USA). Cells were treated with mitochondria‐specific stress reagents, 10 μm gamitrinib‐triphenylphosphonium (GTPP) (a kind gift from D. C. Altieri), 5 μm synthetic triterpenoid 2‐cyano‐3, 12‐dioxooleana‐1, 9(11)‐dien‐28‐oic acid (CDDO) (Cayman Chemicals, Ann Arbor, MI, USA), and 20 μm rotenone (Sigma‐Aldrich, St. Louis, MO, USA) for 6 h.

### Assessment of mRNA

Total RNA was isolated from cells using TRIzol (Ambion, Carlsband, CA, USA). First‐strand cDNA was synthesized using PrimeScript II Reverse Transcriptase and oligo‐dT primer in accordance with the manufacturer's instructions (TAKARA, Kusatsu, Japan). Real‐time quantitative PCR (qPCR) was performed using StepOnePlus (Applied Biosystems, Foster City, CA, USA) with the Power SYBR Green PCR Master Mix (Applied Biosystems) using primers for mouse HSP60 (HSPD1), HSP10 (HSPE1), mtHSP70 (HSPA9), Lon, and HSP70 (HSPA1A and HSPA1B) (Table S1). Relative quantities of mRNAs were normalized against GAPDH or RPLPO (large ribosomal protein) mRNA levels. All reactions were performed in triplicate with samples derived from three experiments.

### RNA interference

To generate adenovirus vectors expressing short hairpin RNAs against mouse HSF1, SSBP1 and TRAP1, oligonucleotides containing each target sequence (Table S2) were annealed and inserted into pCR2.1‐hU6 at the BamHI/HindIII sites, and then, XhoI/HindIII fragments containing hU6‐shRNA were inserted into a pShuttle‐CMV vector (Stratagene) [20]. To knock down HSF1, SSBP1, or TRAP1, MEF cells were infected with Ad‐sh‐mHSF1‐KD, Ad‐sh‐mSSBP1‐KD, or Ad‐sh‐mTRAP1‐KD (1 × 10^8^ pfu·mL^−1^) for 2 h and maintained in normal medium for 70 h. As a control, the cells were infected with an adenovirus vector expressing scrambled RNA (Ad‐sh‐SCR).

### Western blotting

Cells pellets were lysed with NP‐40 buffer (150 mm NaCl, 1.0% Nonidet P‐40, 50 mm Tris/HCl, pH 8.0) containing protease inhibitors (1 mm phenylmethylsulfonyl fluoride, 1 μg·mL^−1^ leupeptin, and 1 μg·mL^−1^ pepstatin) on ice for 10 min. After centrifugation at 16 000 ***g*** for 10 min, supernatants were subjected to SDS/PAGE. For HSP10 blot, a Real Gel Plate with 10‐20% polyacrylamide gel (MDG‐296; BIO CRAFT, Tokyo, Japan) was used. After proteins were transferred onto nitrocellulose or PVDF (SSBP1 blot) membranes, the membranes were blocked in PBS/5% milk at a room temperature for 1 h and then were immunoblotted using rabbit antibodies against HSF1 (anti‐mHSF1j, Millipore ABE1044; dilution, 1 : 1000) [21], TRAP1 (anti‐mTRAP1a; dilution, 1 : 1000) (this study) HSP60 (anti‐HSP60‐1; 1 : 2000) [22], HSP10 (Santa Cruz, CA, USA sc‐20958; 1 : 1000), mtHSP70 (or GRP75) (Santa Cruz sc‐13967; 1 : 1000), and SSBP1 (anti‐mSSBP1x; dilution, 1 : 1000) (this study), and mouse antibody for HSP70 (Santa Cruz W27; 1 : 1000) and β‐actin (AC‐15; Sigma, St. Louis, MO, USA) diluted in PBS/2% milk at a room temperature for 1 h or at 4 °C overnight. The membranes was washed three times with PBS for 5 min and incubated at room temperature for 1 h with secondary antibodies: peroxidase‐conjugated goat anti‐rabbit or anti‐mouse IgG. After washing with PBS/0.1% Tween‐20 three times, chemiluminescent signals from ECL detection reagents (GE Healthcare, Buckinghamshire, UK) were captured on X‐ray film (Super RX; Fujifilm, Tokyo, Japan). Intensity of the bands was quantified using NIH imagej (NIH, Washington, DC, USA). We generated rabbit antisera against mouse TRAP1 (anti‐mTRAP1a) and SSBP1 (anti‐mSSBP1x) by immunizing rabbits using TiterMax Gold adjuvant (CytRx, Los Angeles, CA, USA) with bacterially expressed recombinant GST‐mTRAP1 (full‐length protein) and GST‐mSSBP1 (full‐length protein), respectively.

### Cross‐linking

Mouse embryonic fibroblasts cells were treated with 10 μm GTPP, 5 μm CDDO, or 20 μm rotenone for 6 h, or heat shock at 42 °C for 30 min. Whole‐cell extracts were prepared in buffer C (0.42 m NaCl, 20 mm HEPES/NaOH, pH 7.9, 25% glycerol, 1.5 mm MgCl_2_, 0.2 mm EDTA) containing protease inhibitors [21]. Aliquots containing 40 μg protein were mixed with a 0.05 volume of 100 mm disuccinimidyl glutarate (DSG) (final concentration of 5 mm) at room temperature for 30 min and were subjected to western blotting using HSF1 antibody (anti‐mHSF1j).

### Immunofluorescence

HeLa cells were grown on coated glass coverslips in 35 mm culture dishes for 16 h at 37 °C in 5% CO_2_. Cells were fixed with 100% methanol at −20 °C for 15 min and then washed three times with PBS for 5 min each. Subsequently, they were permeabilized and blocked with PBS/0.1% Triton X‐100/5% goat serum at room temperature for 1 h. After washing with PBS once, the coverslips were incubated with rat monoclonal IgG for HSF1 (10H8, ab61382; Abcam, Cambridge, UK) (1 : 200 dilution) at 4 °C overnight and washed three times with PBS. They were then incubated with FITC‐conjugated goat anti‐rabbit IgG (Cappel) (1 : 200 dilution in PBS/2% milk) or Alexa Flour 546‐conjugated goat anti rat IgG (Molecular Probes, Eugene, OR, USA) (1 : 200 dilution) at room temperature for 1 h. Coverslips were washed three times with PBS for 5 min each and then mounted in a VECTASHIELD with 4′,6‐diamino‐2‐phenylindole (DAPI) mounting medium (Vector Laboratories, Burlingame, CA, USA). High‐resolution (×63 objective magnification) confocal images were taken using LSM510 META confocal microscope (Carl Zeiss, Jena, Germany) and were quantified by using Zen lite software (Carl Zeiss). HSF1 fluorescence signals in a total cell and a nucleus were estimated by measuring the average intensities of pixels by manually tracing cellular periphery and the region stained with DAPI, respectively. Percentage of HSF1 fluorescence signal localized in the nucleus was calculated by normalizing the nuclear signal intensity to total fluorescence intensity from the cell.

### Chromatin immunoprecipitation analysis

ChIP experiments were performed using a kit in accordance with the manufacturer's instructions (EMD Millipore, Burlington, MA, USA). The antibody used for ChIP assays was anti‐mHSF1j. Real‐time qPCR of ChIP‐enriched DNAs in *HSP60*, *mtHSP70*, *HSP70* (*HSPA1A*), and its intergenic region was performed using the primers listed in Table S3. Percentage input was determined by comparing the cycle threshold value of each sample to a standard curve generated from a 5‐point serial dilution of genomic input and compensated by values obtained using normal IgG. IgG‐negative control immunoprecipitations for all sites yielded < 0.05% input. All reactions were performed in triplicate with samples derived from three experiments.

### Measurement of mitochondrial membrane potential and oxygen consumption

Mouse embryonic fibroblast cells, which were infected with Ad‐sh‐mHSF1‐KD or Ad‐sh‐SCR, were seeded into plastic 96 well plates at a density of 5 × 10^4^ cells/well and grown for 16 h. After treatment with each inhibitor for 3 h, the cells were stained with MitoTracker Red CMXRos (Molecular Probes) for 30 min. The wells were washed twice with PBS to remove excess fluorescent dye, and fluorescence signals were measured at 540 nm/615 nm (excitation/emission) using an ARVO X4 multilabel plate reader (PerkinElmer, Inc., Waltham, MA, USA). Alternatively, cells infected with Ad‐sh‐mHSF1‐KD or Ad‐sh‐SCR were grown on glass coverslips in 35 mm culture dishes for 16 h, treated as described above, and were fixed with 100% methanol at −20 °C for 15 min. Coverslips were washed three times with PBS for 5 min each and then mounted in a VECTASHIELD with DAPI mounting medium (Vector Laboratories). High‐resolution (×63 objective magnification) confocal images were taken using LSM510 META confocal microscope (Carl Zeiss).

Oxygen consumption was examined by using MitoXpress Xtra Oxygen Consumption Assay (Agilent, Chicopee, MA, USA) in accordance with the manufacturer's instructions. MEF cells were treated as described above in plastic 96 well plates and maintained at 37 °C on a thermoregulator. The cells were loaded with a reagent containing the oxygen‐sensitive MitoXpress Xtra fluorescent probe and treated with or without 500 nm FCCP (Cayman Chemical) or 5 μm antimycin A (Abcam), and were covered by mineral oil. Each sample well was then measured at 340 nm/642 nm (excitation/emission) repetitively every 5 min over 120 min using an ARVO X4 multilabel plate reader (PerkinElmer, Inc.), by taking TR‐F intensity readings at delay time of 30 and 70 μs and gate time 100 μs. Measured TR‐F intensity signals (counts·s^−1^) were converted into lifetime signals (μs). Relative oxygen consumption rate (OCR) was estimated as a value of MitoXpress Xtra fluorescence lifetime signal per hour per mg of protein (μs·h^−1^·mg^−1^).

### Statistical analysis

Data were analyzed using Student's *t*‐test for comparisons between two groups. Multiple‐group differences were assessed by one‐way ANOVA test, followed by the Tukey *post hoc* test (jmp pro 14 software; SAS Institute Inc., Cary, NC, USA). Asterisks in figures indicate that differences were significant (*P* < 0.01 or 0.05). Error bars represent the standard deviations for more than three independent experiments.

## Results

### HSF1 is required for activation of mitochondrial chaperone genes

**Fig. 1:**
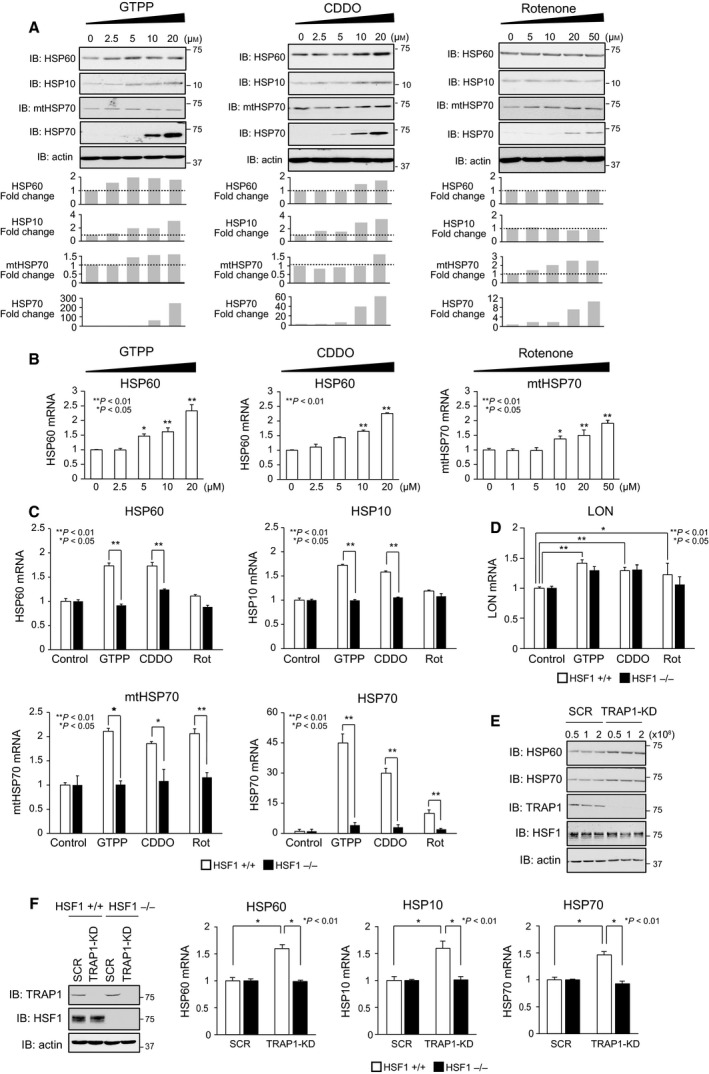
HSF1 is required for the activation of mitochondrial chaperone genes. (A) Induction of mitochondrial chaperones during treatment with reagents that target mitochondrial proteins. MEF cells were treated with GTPP, CDDO, or rotenone at the indicated concentrations for 6 h. Cell extracts were prepared using NP‐40 lysis buffer and were subjected to western blotting (upper). Intensity of HSP bands in representative blots was quantified using NIH imagej and normalized to the intensity of each actin loading control. Fold changes during treatments are shown (lower). (B) Induction of HSP60 and mtHSP70 mRNAs during treatment with reagents that target mitochondrial proteins. mRNA levels were quantified by RT‐qPCR (*n* = 3). Mean ± SD is shown. Asterisks indicate *P* < 0.01 or 0.05 by one‐way ANOVA, compared with each mRNA level in untreated cells. (C) HSF1 is required for the induction of mitochondrial HSP mRNAs in cells treated with the indicated reagents. Wild‐type (HSF1+/+) and HSF1‐null (HSF1−/−) MEF cells were treated with 10 μm GTPP, 5 μm CDDO, and 20 μm rotenone for 6 h. mRNA levels of HSP60, HSP10, mtHSP70, and HSP70 were quantified by RT‐qPCR (*n* = 3). Mean ± SD is shown. Asterisks indicate *P* < 0.01 or 0.05 by Student's *t*‐test. (D) HSF1 is not required for the induction of Lon mRNAs in cells treated with the indicated reagents. Cells were treated as is shown in B. mRNA levels of Lon were quantified by RT‐qPCR (*n* = 3). Mean ± SD is shown. Asterisks indicate *P* < 0.01 or 0.05 by Student's *t*‐test. (E) HSP60 is induced by TRAP1 knockdown. MEF cells were infected with an adenovirus expressing scrambled RNA (SCR) or shRNA for TRAP1 (TRAP1‐KD) at the indicated concentration (0.5 to 2 × 10^8^ PFU·mL^−1^) for 2 h, maintained with normal medium for 70 h. Cell extracts were prepared and subjected to western blotting. (F) HSF1 is required for the induction of HSP60 and HSP10 mRNAs in TRAP1‐knockdown cells. Wild‐type (HSF1+/+) and HSF1‐null (HSF1−/−) MEF cells were infected with each adenovirus (1 × 10^8^ PFU·mL^−1^) as described in (E). mRNA levels of HSP60, HSP10, and HSP70 were quantified by RT‐qPCR (*n* = 3). Mean ± SD is shown (right). Asterisks indicate *P* < 0.01 by Student's *t*‐test. Cell extracts were prepared and subjected to western blotting (left).

To examine the roles of HSF1 in the UPR^mt^, we treated immortalized MEF cells with three reagents that target mitochondrial proteins and impair mitochondrial proteostasis. GTPP inhibits the matrix HSP90 chaperone TRAP1 [23, 24], and CDDO inhibits the matrix protease Lon [25]. Rotenone is an inhibitor of the electron transfer complex 1 (ETC1) and increases production of reactive oxygen species (ROS). Protein levels of HSP60 and HSP10 were increased by treatment with GTPP or CDDO at concentrations of 5–20 μm, but were not by treatment with rotenone (Fig. 1A). In contrast, mtHSP70 protein levels were increased by treatment with 10–50 μm rotenone and were slightly increased by treatment with GTPP or CDDO. HSP60 mRNA levels were also increased by treatment with GTPP or CDDO, and mtHSP70 mRNA levels were increased by treatment with rotenone (Fig. 1B). Thus, the treatment of MEF cells with these reagents induced at least some mitochondrial HSPs in a dose‐dependent manner, as reported previously [26, 27]. We then treated wild‐type and HSF1‐null MEF cells with 10 μm GTPP, 5 μm CDDO, or 20 μm rotenone for 6 h and found that HSP60, HSP10, and mtHSP70 mRNA levels were increased by 1.2‐ to 2.0‐fold in wild‐type cells treated with GTPP and CDDO, and only mtHSP70 mRNA levels were significantly increased in cells treated with rotenone (Fig. 1C). Remarkably, mRNA levels of these genes were not induced in HSF1‐null cells at all. mRNA levels of HSP70 were simultaneously increased by 10‐ to 45‐fold in wild‐type cells in a HSF1‐dependent manner, suggesting that cytoplasmic proteostasis was also impaired in these conditions (Fig. 1C) [28]. In marked contrast, expression of mitochondrial protease Lon mRNA was induced in both wild‐type and HSF1‐null cells during the treatment (Fig. 1D). To exclude nonspecific effects of GTPP, we knocked down TRAP1 and confirmed that both HSP60 and HSP70 protein levels were increased in TRAP1‐knockdown cells (Fig. 1E) [27, 29]. HSP60, HSP10, and HSP70 mRNA levels were also increased by about 1.5‐fold (Fig. 1F). However, they were not increased at all in TRAP1‐knockdown cells deficient in HSF1. These results demonstrated that HSF1 is required for activation of mitochondrial chaperone genes, but not for that of *Lon* protease gene, during the UPR^mt^ in mouse cells, when mitochondrial proteostasis is impaired by targeting a mitochondrial chaperone or protease, or an ETC component.

### Different requirement of SSBP1 for activation of the mitochondrial chaperone genes

**Fig. 2:**
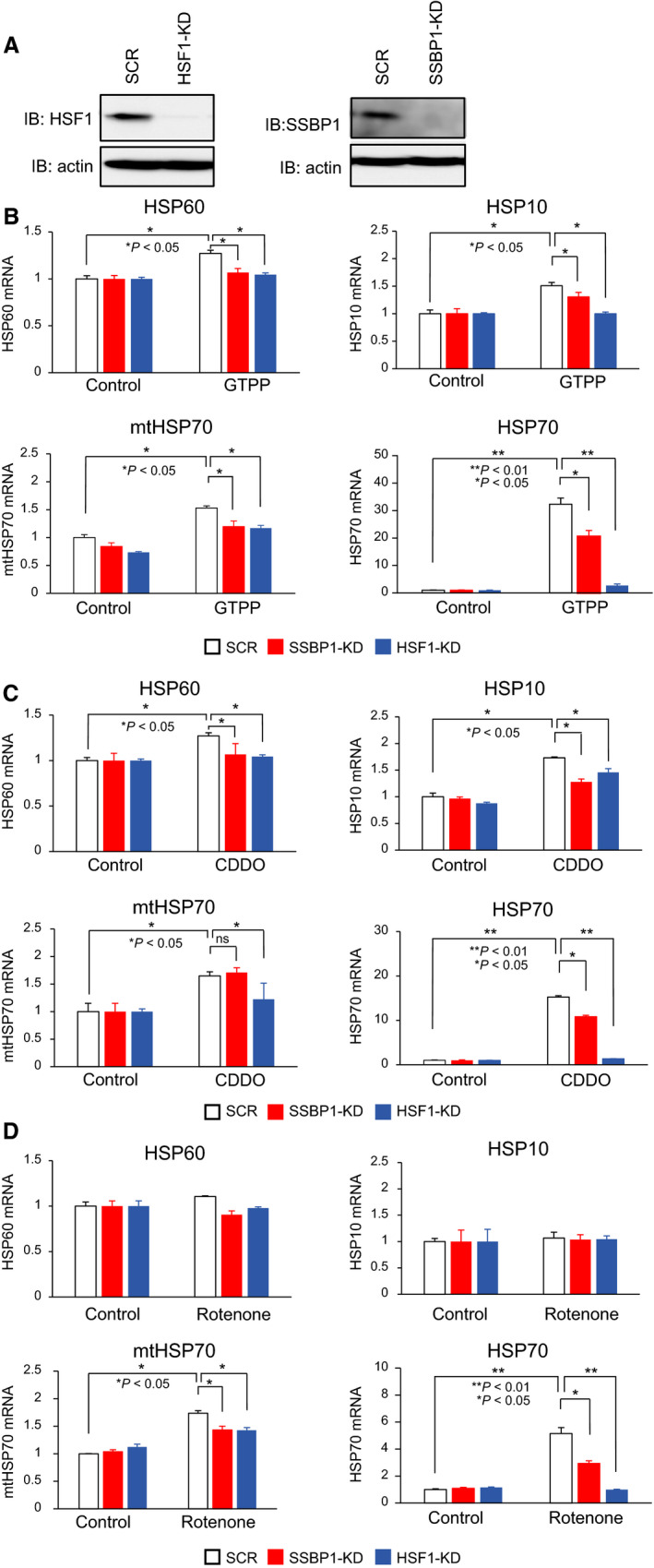
Different requirements of SSBP1 for the activation of mitochondrial chaperone genes. (A) Knockdown of HSF1 or SSBP1. MEF cells were infected with an adenovirus expressing scrambled RNA (SCR) or shRNA for SSBP1 (SSBP1‐KD) or HSF1 (HSF1‐KD) for 2 h, maintained with normal medium for 70 h. Cell extracts were prepared and subjected to western blotting. (B–D) Activation of the mitochondrial chaperone genes in SSBP1‐ or HSF1‐knockdown cells. SSBP1 or HSF1 was knocked down as described in (A). The cells were treated with 10 μm GTPP (B), 5 μm CDDO (C), and 20 μm rotenone (D) for 6 h. mRNA levels of HSP60, HSP10, mtHSP70, and HSP70 were quantified by RT‐qPCR (*n* = 3). Mean ± SD is shown. Asterisks indicate *P* < 0.01 or 0.05 by Student's *t*‐test (ns, not significant).

We then investigated the effects of SSBP1 on the activation of UPR^mt^ genes in response to impaired mitochondrial proteostasis. MEF cells were infected for 72 h with an adenovirus expressing short hairpin RNA for SSBP1 or HSF1, or scrambled RNA (SCR) as a control, and protein level of SSBP1 or HSF1 was transiently reduced (Fig. 2A). We confirmed that the expression of HSP60, HSP10, and mtHSP70 mRNAs as well as HSP70 mRNA was hardly increased in HSF1‐knockdown cells during treatment with GTPP, CDDO, or rotenone (Fig. 2B–D, black bars). In SSBP1‐knockdown cells, the expression of HSP70 mRNA was partially increased during the same treatment (Fig. 2B–D, gray bars), like during treatment with heat shock [18]. In marked contrast, HSP60 mRNA expression was not increased at all in SSBP1‐knockdown cells during treatment with GTPP or CDDO. Similarly, HSP10 mRNA expression was less increased in SSBP1‐knockdown cells during GTPP and CDDO treatment than scrambled RNA‐treated cells (Fig. 2B,C, gray bars). On the other hand, mtHSP70 mRNA expression was fully increased in SSBP1‐knockdown cells during CDDO treatment, whereas it was less increased in the same cells treated with GTPP or rotenone (Fig. 2B–D, gray bars). These results suggested different requirements of SSBP1 on the activation of mitochondrial chaperone genes during the UPR^mt^.

### Nuclear translocation, trimer formation, and phosphorylation of HSF1

**Fig. 3:**
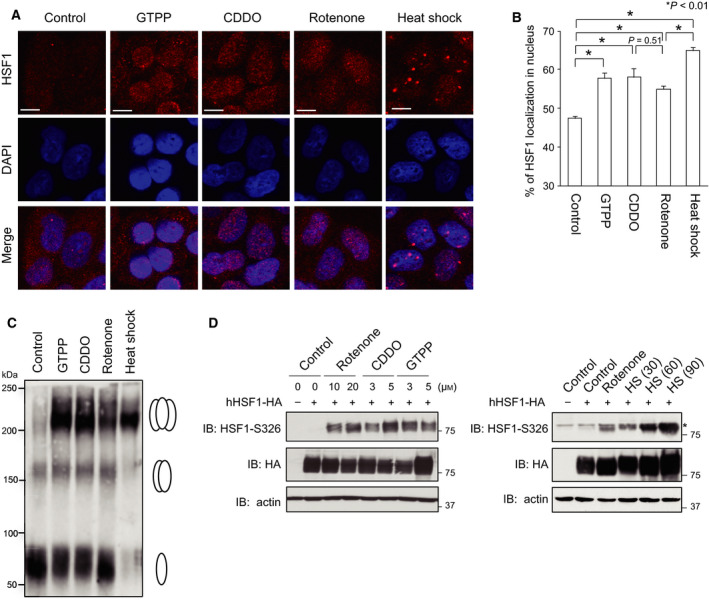
HSF1 is moderately activated in response to impaired mitochondrial proteostasis. (A) Nuclear translocation of HSF1 in response to impaired mitochondrial proteostasis. HeLa cells were treated with 10 μm GTPP, 5 μm CDDO, or 20 μm rotenone for 6 h. Some cells were also treated with heat shock at 42 °C for 30 min. The cells were costained with an antibody for HSF1 and the nuclear marker DAPI, and fluorescence images were merged (Merge). Bars, 20 μm. (B) Quantitative estimation of HSF1 signals in the nucleus. Fluorescence signals in the nucleus and total fluorescence signals from the cell were estimated (*n* = 20), and percentages of HSF1 localized in nucleus are shown. Mean ± SD is shown. Asterisks indicate *P* < 0.01 by one‐way ANOVA, compared with the percentage in control cells. (C) Trimer formation of HSF1. MEF cells were treated with 10 μm GTPP, 5 μm CDDO, or 20 μm rotenone for 6 h, or heat shock at 42 °C for 30 min. Whole‐cell extracts were prepared, and aliquots containing 40 μg protein were mixed with a cross‐linking reagent DSG at a final concentration of 5 mm at room temperature for 30 min and were subjected to western blotting using HSF1 antibody. Positions of HSF1 monomer, dimer, and trimer are indicated on the right. (D) Phosphorylation of HSF1‐Ser326. HSF1‐null (HSF1−/−) cells were infected with adenovirus expressing HA‐tagged human HSF1 (1 × 10^8^ pfu·mL^−1^) for 2 h and maintained with normal medium for 46 h. The cells were treated with GTPP, CDDO, or rotenone at the indicated concentrations for 6 h (left), or treated with 20 μm rotenone for 6 h or heat shock (HS) at 42 °C for 30, 60, or 90 min (right). Cell extracts were prepared and subjected to western blotting using HSF1‐phospho‐S326 (S326P), HA, or β‐actin antibody. An asterisk indicates nonspecific bands.

We investigated whether HSF1 is activated directly or indirectly during treatment with GTPP, CDDO, or rotenone. HSF1 activation involves its nuclear translocation, trimer formation, and phosphorylation of a specific residue [30, 31]. First, we performed immunofluorescence analysis of HeLa cells using confocal microscopy because HSF1 localization was intensively studied in the cells [18]. We found that HSF1 localizes to both the cytoplasm and nucleus in unstressed cells and slightly accumulates in the nucleus during treatment with GTPP, CDDO, or rotenone (Fig. 3A,B). Nuclear foci termed HSF1 granules were detected in cells treated with heat shock but not in cells treated with these reagents. Second, we examined the oligomeric form of HSF1 using DSG cross‐linking experiments. Monomeric HSF1 shifted to a trimeric form during treatment of MEF cells with heat shock and was partly shifted to a trimeric form during treatment with GTPP, CDDO, or rotenone (Fig. 3C). Third, we studied HSF1‐Ser326 phosphorylation, which is an active mark of HSF1 transcriptional activity [32]. Because a specific antibody for human HSF1‐Ser326, but not for mouse HSF1‐Ser326, is available, we replaced endogenous HSF1 with human HSF1 in MEF cells. It was revealed that hHSF1‐Ser326 was phosphorylated at lower levels in cells treated with GTPP, CDDO, or rotenone than in cells treated with heat shock at 42 °C 90 min (Fig. 3D). Hyperphosphorylation of HSF1, which is detected as retarded bands on a gel, is often correlated with the activation of HSF1, but was not evident in the same cells. These results suggested that HSF1 is modestly activated in response to impaired mitochondrial proteostasis.

### HSF1 occupancy in HSP60/HSP10 promoter is remarkably high

**Fig. 4:**
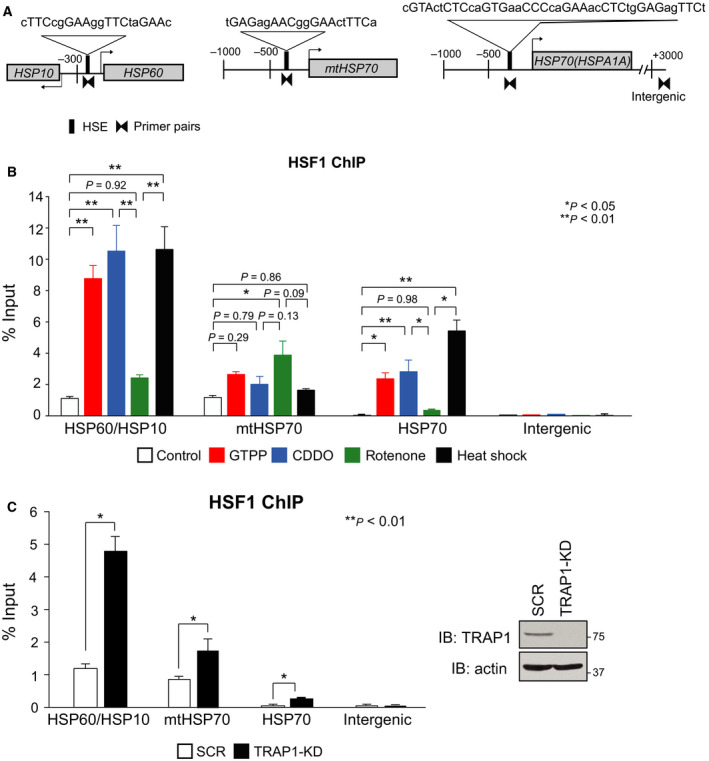
HSF1 occupancy on the mitochondrial chaperone gene promoters is induced at different levels. (A) Schematic representation of mouse *HSP60/HSP10*, *mtHSP70*, and *HSP70 (HSPA1A)* loci. Nucleotide sequences of each HSE and consensus nGAAn sequences are shown. Amplified promoter regions by qPCR using each specific primer pairs are indicated. (B) HSF1 occupancy is induced in response to impaired mitochondrial proteostasis. MEF cells were treated with 10 μm GTPP, 5 μm CDDO, 20 μm rotenone for 6 h, or heat shock at 42 °C for 30 min. ChIP‐qPCR performed on each promoter region using HSF1 antibody (*n* = 3). Mean ± SD is shown. Asterisks indicate *P* < 0.01 or 0.05 by one‐way ANOVA, compared between two groups. (C) TRAP1 knockdown enhances HSF1 occupancy on *HSP60/HSP10* promoter. MEF cells were infected for 72 h with Ad‐sh‐mTRAP1‐KD, and ChIP‐qPCR analyses were performed using HSF1 antibody. Mean ± SD is shown (*n* = 3) (left). Asterisks indicate *P* < 0.01 by Student's *t*‐test. Cell extracts were prepared and subjected to western blotting (right).

It was assumed that HSF1 mildly occupies mitochondrial chaperone gene promoters *in vivo* in impaired mitochondrial proteostasis conditions, because it is activated only modestly. As shown previously, HSF1 heavily bound to *HSP60/HSP10* promoter as well as *HSP70* (*HSPA1A*) promoter, and a little to *mtHSP70* promoter during heat shock at 42 °C for 30 min (Fig. 4A,B). In contrast, HSF1 moderately bound to *HSP70* promoter in cells treated with GTPP and CDDO and bound to it at a lower level in cells treated with rotenone. HSF1 constitutively bound to *mtHSP70* promoter to some extent, and its binding was induced moderately in cells treated with rotenone and was little induced in cells treated with GTPP and CDDO. HSF1 also constitutively bound to *HSP60/HSP10* promoter to some extent, and the levels of HSF1 binding were little induced in cells treated with rotenone. Contrary to our expectation, the levels of HSF1 binding were heavily induced in cells treated with GTPP and CDDO, like in cells treated with heat shock (Fig. 4A,B). Furthermore, we confirmed that levels of HSF1 binding to *HSP60/HSP10* promoter were markedly induced in TRAP1‐knockdown cells (Fig. 4C). These results indicated that HSF1 occupancy on the mitochondrial chaperone gene promoters is induced at different levels. HSF1 occupancy in *HSP60/HSP10* promoter was remarkably high during the treatment with GTPP and CDDO, whereas that in *mtHSP70* or *HSP70* promoter was moderate.

### HSF1 promotes the maintenance of mitochondrial function

**Fig. 5:**
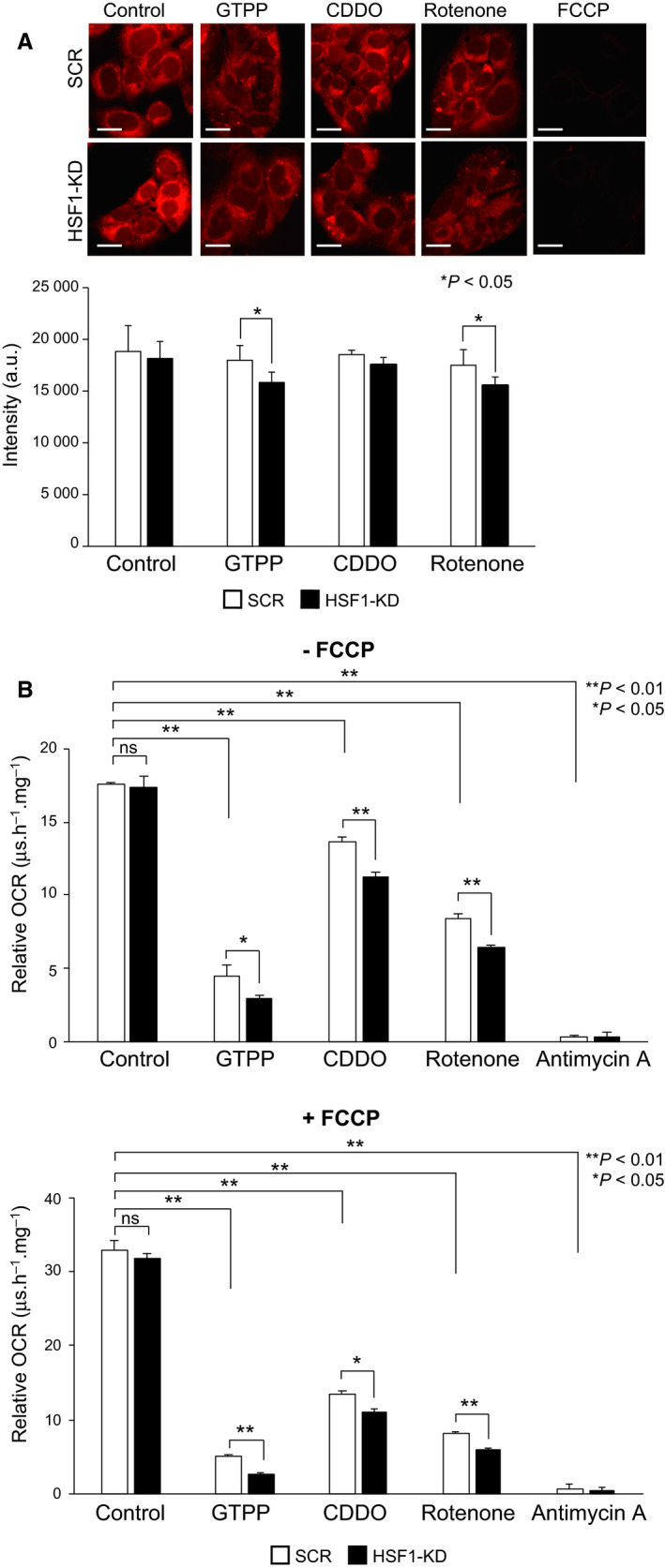
HSF1 is involved in the maintenance of mitochondrial function. (A) HSF1 supports the maintenance of mitochondrial membrane potential. Control (SCR) and HSF1‐knockdown (HSF1‐KD) MEF cells were treated with 10 μm GTPP, 5 μm CDDO, or 20 μm rotenone for 3 h or treated with 10 μm FCCP for 20 min as a control. The cells were stained with MitoTracker Red CMXRos (upper), and MitoTracker fluorescent signals were measured (arbitrary fluorescence unit) (lower). Mean ± SD is shown (*n* = 3). Asterisks indicate *P* < 0.05 by Student's *t*‐test. Bars, 20 μm. (B) HSF1 supports the maintenance of oxygen consumption. Control (SCR) and HSF1‐knockdown (HSF1‐KD) MEF cells were treated with 10 μm GTPP, 5 μm CDDO, or 20 μm rotenone for 3 h and mixed with MitoXpress Xtra reagent containing an oxygen‐sensitive fluorescence probe. Fluorescent signals were measured in the presence (lower) or absence (upper) of 500 nm FCCP. Fluorescent signals in the presence of 5 μm antimycin A were measured as a control. Relative oxygen consumption rate (OCR) was estimated as a value of MitoXpress Xtra fluorescence lifetime signal per hour per mg of protein (μs·h^−1^·mg^−1^). Mean ± SD is shown (*n* = 3). Asterisks indicate *P* < 0.01 or 0.05 by Student's *t*‐test.

To test whether HSF1‐mediated expression of UPR^mt^ genes is related with mitochondrial function, we first examined mitochondrial membrane potential using a fluorescent probe MitoTracker Red. The intensity of MitoTracker fluorescence was not affected when MEF cells were treated with 10 μm GTPP, 5 μm CDDO, or 20 μm rotenone for 3 h (Fig. 5A). However, it was significantly reduced in HSF1‐knockdown cells treated with GTPP or rotenone, but not in those cells treated with CDDO. We next examined the basal (−FCCP) and maximal (+FCCP) oxygen consumption in the same cells (Fig. 5B). The relative oxygen consumption rate (OCR) was not significantly reduced by HSF1 knockdown, but was reduced in cells treated with GTPP, CDDO, or rotenone for 3 h. Remarkably, the levels of basal and maximal respiration in the presence of GTPP, CDDO, or rotenone were more reduced in HSF1‐knockdown cells than those in scrambled RNA‐treated cells. These results suggested that HSF1 promotes the maintenance of mitochondrial function in response to impaired mitochondrial proteostasis.

## Discussion

Mitochondria are the central hub of metabolic and signaling processes including ATP production and apoptotic cell death [33, 34], and declines in mitochondrial function are associated with aging and disorders, such as neurodegenerative diseases and cancer [35, 36]. Cells must adapt to a large variety of mitochondrial dysfunctions by changing nuclear‐encoded mitochondrial gene expression. Among these homeostatic mechanisms, the UPR^mt^ is an adaptive response to accumulation of misfolded proteins in mitochondria. ATF5 and CHOP have been shown to be required for the activation of UPR^mt^ genes during accumulation of ΔOTC in human HEK293 and monkey COS‐7 cells, respectively [7, 13]. In this study, we used immortalized MEF cells for analysis of the UPR^mt^, and mechanisms of the UPR^mt^ were analyzed during treatment with GTPP, CDDO, or rotenone [26, 27, 37], which induces the expression of HSP60, HSP10, mtHSP70, or Lon as well as cytoplasmic HSP70. We showed that both disruption of *HSF1* gene and transient HSF1 knockdown abolished the upregulation of mitochondrial chaperone genes, but not for that of protease *Lon*, during the UPR^mt^ (Figs 1 and 2). In contrast, SSBP1 is required for the upregulation of only *HSP60*. Even in unstressed conditions, HSF1 constitutively occupied *HSP60/HSP10* and *mtHSP70* promoters (Fig. 4). Furthermore, a very small part of HSF1 accumulated in the nucleus, shifted to a trimeric form, and was phosphorylated at Ser326, suggesting that HSF1 was activated directly or indirectly in response to impaired mitochondrial proteostasis (Fig. 3). Although treatment with the inhibitors may also cause proteostasis impairment in the cytoplasm, our observation indicated that HSF1 is required for activation of mitochondrial chaperone genes during the UPR^mt^.

HSF1 has been shown to plays roles in the maintenance of mitochondrial function through different pathways. HSF1 deficiency causes reduced constitutive expression of cytoplasmic HSPs including HSP25, which is associated with a decrease in cellular GSH/GSSG ratio and an increase in mitochondrial oxidative stress in the heart, kidney, and oocytes [38, 39, 40, 41]. Induction of HSPs, including HSP60 and HSP10, by HSF1 and SSBP1 promotes the maintenance of mitochondrial membrane potential in proteotoxic stress conditions, which are caused by heat shock or proteasome inhibition [18]. Furthermore, activation of HSF1 is associated with increased mitochondrial function by enhancing the expression of PGC1α, which is a central regulator of mitochondrial biogenesis and function [42]. Consistently, mitochondrial function such as mitochondrial membrane potential is suggested to be more reduced by the expression of an aggregation‐prone polyglutamine protein in HSF1‐knockdown cells [43]. Here, we showed that mitochondrial membrane potential or relative OCR were more reduced in HSF1‐knockdown cells than those in scrambled RNA‐treated cells during treatment with GTPP, CDDO, or rotenone (Fig. 5). Our observations suggested that mitochondrial function in conditions of impaired mitochondrial proteostasis is maintained in part by the HSF1‐dependent upregulation of mitochondrial chaperone genes.

It is worth noting that *HSP60* and *HSP10* uniquely share a bidirectional promoter containing an HSE, which consisted of at least four inverted repeats of an exceptionally conserved consensus nGAAn unit [16, 17]. ChIP‐seq and ChIP‐qPCR data analysis showed that HSF1 constitutively binds to the bidirectional promoter at a much higher level than to the promoters of other *HSP* genes including *HSP70* in MEF cells, and the level of HSF1 binding to this promoter was dramatically elevated during heat shock [18, 19]. HSF1 was mostly converted to a DNA‐binding trimer during heat shock, whereas a small part of HSF1 shifted to a trimer during the UPR^mt^ (Fig. 3C). Unexpectedly, *in vivo* HSF1 binding to the bidirectional promoter was induced in cells treated with GTPP and CDDO at the same levels as that in cells treated with heat shock (Fig. 4), although level of HSF1 binding to this promoter was little elevated in cells treated with rotenone. Thus, analysis of *in vivo* HSF1 binding to the unique bidirectional promoter of *HSP60/HSP10* could be a sensitive marker of the UPR^mt^.

## Conflict of interest

The authors declare no conflict of interest.

## Author contributions

AK, MF, and AN designed the research. AK, MF, KT, AK, PS, MO, and RT performed experiments. AK and AN wrote the manuscript. All authors discussed the results and commented on the manuscript.

## Supporting information


**Table S1.** Primer sequences used for RT‐qPCR.
**Table S2.** Nucleotide sequences of shRNAs used for gene knockdown.
**Table S3.** Primer sequences used for ChIP assay.

## References

[1] Wolff S , Weissman JS and Dillin A (2014) Differential scales of protein quality control. Cell 157, 52–64. 24679526 10.1016/j.cell.2014.03.007

[2] Morimoto RI (1998) Regulation of the heat shock transcriptional response: cross talk between a family of heat shock factors, molecular chaperones, and negative regulators. Genes Dev 12, 3788–3796. 9869631 10.1101/gad.12.24.3788

[3] Gomez‐Pastor R , Burchfiel ET and Thiele DJ (2018) Regulation of heat shock transcription factors and their roles in physiology and disease. Nat Rev Mol Cell Biol 19, 4–19. 28852220 10.1038/nrm.2017.73 PMC5794010

[4] Ron D and Walter P (2007) Signal integration in the endoplasmic reticulum unfolded protein response. Nat Rev Mol Cell Biol 8, 519–529. 17565364 10.1038/nrm2199

[5] Haynes CM and Ron D (2010) The mitochondrial UPR – protecting organelle protein homeostasis. J Cell Sci 123, 3849–3855. 21048161 10.1242/jcs.075119

[6] Martinus RD , Garth GP , Webster TL , Cartwright P , Naylor DJ , Høj PB and Hoogenraad NJ (1996) Selective induction of mitochondrial chaperones in response to loss of the mitochondrial genome. Eur J Biochem 240, 98–103. 8797841 10.1111/j.1432-1033.1996.0098h.x

[7] Zhao Q , Wang J , Levichkin IV , Stasinopoulos S , Ryan MT and Hoogenraad NJ (2002) A mitochondrial specific stress response in mammalian cells. EMBO J 21, 4411–4419. 12198143 10.1093/emboj/cdf445 PMC126185

[8] Yoneda T , Benedetti C , Urano F , Clark SG , Harding HP and Ron D (2004) Compartment‐specific perturbation of protein handling activates genes encoding mitochondrial chaperones. J Cell Sci 117, 4055–4066. 15280428 10.1242/jcs.01275

[9] Nargund AM , Pellegrino MW , Fiorese CJ , Baker BM and Haynes CM (2012) Mitochondrial import efficiency of ATFS‐1 regulates mitochondrial UPR activation. Science 337, 587–590. 22700657 10.1126/science.1223560 PMC3518298

[10] Quirós PM , Mottis A and Auwerx J (2016) Mitonuclear communication in homeostasis and stress. Nat Rev Mol Cell Biol 17, 213–226. 26956194 10.1038/nrm.2016.23

[11] Melber A and Haynes CM (2018) UPRmt regulation and output: a stress response mediated by mitochondrial‐nuclear communication. Cell Res 28, 281–295. 29424373 10.1038/cr.2018.16 PMC5835775

[12] Merkwirth C , Jovaisaite V , Durieux J , Matilainen O , Jordan SD , Quiros PM , Steffen KK , Williams EG , Mouchiroud L , Tronnes SU *et al* (2016) Two conserved histone demethylases regulate mitochondrial stress‐induced longevity. Cell 165, 1209–1223. 27133168 10.1016/j.cell.2016.04.012 PMC4889222

[13] Fiorese CJ , Schulz AM , Lin YF , Rosin N , Pellegrino MW and Haynes CM (2016) The transcription factor ATF5 mediates a mammalian mitochondrial UPR. Curr Biol 26, 2037–2043. 27426517 10.1016/j.cub.2016.06.002 PMC4980197

[14] Horibe T and Hoogenraad NJ (2007) The chop gene contains an element for the positive regulation of the mitochondrial unfolded protein response. PLoS ONE 2, e835. 17848986 10.1371/journal.pone.0000835 PMC1950685

[15] Mizzen LA , Chang C , Garrels JI and Welch WJ (1989) Identification, characterization, and purification of two mammalian stress proteins present in mitochondria, grp 75, a member of the hsp 70 family and hsp 58, a homolog of the bacterial groEL protein. J Biol Chem 264, 20664–20675. 2573603

[16] Ryan MT , Herd SM , Sberna G , Samuel MM , Hoogenraad NJ and Høj PB (1997) The genes encoding mammalian chaperonin 60 and chaperonin 10 are linked head‐to‐head and share a bidirectional promoter. Gene 196, 9–17. 9322735 10.1016/s0378-1119(97)00111-x

[17] Hansen JJ , Bross P , Westergaard M , Nielsen M , Eiberg H , Børglum AD , Mogensen J , Kristiansen K , Bolund L and Gregersen N (2003) Genomic structure of the human mitochondrial chaperonin genes: HSP60 and HSP10 are localised head to head on chromosome 2 separated by a bidirectional promoter. Hum Genet 112, 436. 12483302 10.1007/s00439-002-0837-9

[18] Tan K , Fujimoto M , Takii R , Takaki E , Hayashida N and Nakai A (2015) Mitochondrial SSBP1 protects cells from proteotoxic stresses by potentiating stress‐induced HSF1 transcriptional activity. Nat Commun 6, 6580. 25762445 10.1038/ncomms7580 PMC4558571

[19] Takii R , Fujimoto M , Tan K , Takaki E , Hayashida N , Nakato R , Shirahige K and Nakai A (2015) ATF1 modulates the heat shock response by regulating the stress‐inducible HSF1‐transcription complex. Mol Cell Biol 35, 11–25. 25312646 10.1128/MCB.00754-14 PMC4295370

[20] Fujimoto M , Takii R , Katiyar A , Srivastava P and Nakai A (2018) Poly(ADP‐ribose) polymerase 1 promotes the human heat shock response by facilitating heat shock transcription factor 1 binding to DNA. Mol Cell Biol 38, e00051‐18. 29661921 10.1128/MCB.00051-18 PMC6002698

[21] Fujimoto M , Takaki E , Takii R , Tan K , Prakasam R , Hayashida N , Iemura S , Natsume T and Nakai A (2012) RPA assists HSF1 access to nucleosomal DNA by recruiting histone chaperone FACT. Mol Cell 48, 182–194. 22940245 10.1016/j.molcel.2012.07.026

[22] Fujimoto M , Izu H , Seki K , Fukuda K , Nishida T , Yamada S , Kato K , Yonemura S , Inouye S and Nakai A (2004) HSF4 is required for normal cell growth and differentiation during mouse lens development. EMBO J 23, 4297–4306. 15483628 10.1038/sj.emboj.7600435 PMC524399

[23] Kang BH , Plescia J , Song HY , Meli M , Colombo G , Beebe K , Scroggins B , Neckers L and Altieri DC (2009) Combinatorial drug design targeting multiple cancer signaling networks controlled by mitochondrial Hsp90. J Clin Invest 119, 454–464. 19229106 10.1172/JCI37613 PMC2648691

[24] Siegelin MD , Dohi T , Raskett CM , Orlowski GM , Powers CM , Gilbert CA , Ross AH , Plescia J and Altieri DC (2011) Exploiting the mitochondrial unfolded protein response for cancer therapy in mice and human cells. J Clin Invest 121, 1349–1360. 21364280 10.1172/JCI44855 PMC3069780

[25] Bernstein SH , Venkatesh S , Li M , Lee J , Lu B , Hilchey SP , Morse KM , Metcalfe HM , Skalska J , Andreeff M *et al* (2012) The mitochondrial ATP‐dependent Lon protease: a novel target in lymphoma death mediated by the synthetic triterpenoid CDDO and its derivatives. Blood 119, 3321–3329. 22323447 10.1182/blood-2011-02-340075 PMC3321858

[26] Runkel ED , Liu S , Baumeister R and Schulze E (2013) Surveillance‐activated defenses block the ROS‐induced mitochondrial unfolded protein response. PLoS Genet 9, e1003346. 23516373 10.1371/journal.pgen.1003346 PMC3597513

[27] Münch C and Harper JW (2016) Mitochondrial unfolded protein response controls matrix pre‐RNA processing and translation. Nature 534, 710–713. 27350246 10.1038/nature18302 PMC4939261

[28] Kim HE , Grant AR , Simic MS , Kohnz RA , Nomura DK , Durieux J , Riera CE , Sanchez M , Kapernick E , Wolff S *et al* (2016) Lipid biosynthesis coordinates a mitochondrial‐to‐cytosolic stress response. Cell 166, 1539–1552. 27610574 10.1016/j.cell.2016.08.027 PMC5922983

[29] Baqri RM , Pietron AV , Gokhale RH , Turner BA , Kaguni LS , Shingleton AW , Kunes S and Miller KE (2014) Mitochondrial chaperone TRAP1 activates the mitochondrial UPR and extends health span in *Drosophila* . Mech Ageing Dev 141–142, 35–45. 10.1016/j.mad.2014.09.002 PMC4310785 25265088

[30] Sarge KD , Murphy SP and Morimoto RI (1993) Activation of heat shock gene transcription by heat shock factor 1 involves oligomerization, acquisition of DNA‐binding activity, and nuclear localization and can occur in the absence of stress. Mol Cell Biol 13, 1392–1407. 8441385 10.1128/mcb.13.3.1392-1407.1993 PMC359449

[31] Baler R , Dahl G and Voellmy R (1993) Activation of human heat shock genes is accompanied by oligomerization, modification, and rapid translocation of heat shock transcription factor HSF1. Mol Cell Biol 13, 2486–2496. 8455624 10.1128/mcb.13.4.2486-2496.1993 PMC359569

[32] Guettouche T , Boellmann F , Lane WS and Voellmy R (2005) Analysis of phosphorylation of human heat shock factor 1 in cells experiencing a stress. BMC Biochem 6, 4. 15760475 10.1186/1471-2091-6-4 PMC1079796

[33] Pagliarini DJ and Rutter J (2013) Hallmarks of a new era in mitochondrial biochemistry. Genes Dev 27, 2615–2627. 24352419 10.1101/gad.229724.113 PMC3877752

[34] Friedman JR and Nunnari J (2014) Mitochondrial form and function. Nature 505, 335–343. 24429632 10.1038/nature12985 PMC4075653

[35] Vafai SB and Mootha VK (2012) Mitochondrial disorders as windows into an ancient organelle. Nature 491, 374–383. 23151580 10.1038/nature11707

[36] Campisi J , Kapahi P , Lithgow GJ , Melov S , Newman JC and Verdin E (2019) From discoveries in ageing research to therapeutics for healthy ageing. Nature 571, 183–192. 31292558 10.1038/s41586-019-1365-2 PMC7205183

[37] Quirós PM , Prado MA , Zamboni N , D'Amico D , Williams RW , Finley D , Gygi SP and Auwerx J (2017) Multi‐omics analysis identifies ATF4 as a key regulator of the mitochondrial stress response in mammals. J Cell Biol 216, 2027–2045. 28566324 10.1083/jcb.201702058 PMC5496626

[38] Yan LJ , Christians ES , Liu L , Xiao X , Sohal RS and Benjamin IJ (2002) Mouse heat shock transcription factor 1 deficiency alters cardiac redox homeostasis and increases mitochondrial oxidative damage. EMBO J 21, 5164–5172. 12356732 10.1093/emboj/cdf528 PMC129050

[39] Yan LJ , Rajasekaran NS , Sathyanarayanan S and Benjamin IJ (2005) Mouse HSF1 disruption perturbs redox state and increases mitochondrial oxidative stress in kidney. Antioxid Redox Signal 7, 465–471. 15706094 10.1089/ars.2005.7.465

[40] Metchat A , Akerfelt M , Bierkamp C , Delsinne V , Sistonen L , Alexandre H and Christians ES (2009) Mammalian heat shock factor 1 is essential for oocyte meiosis and directly regulates Hsp90alpha expression. J Biol Chem 284, 9521–9528. 19158073 10.1074/jbc.M808819200 PMC2666604

[41] Bierkamp C , Luxey M , Metchat A , Audouard C , Dumollard R and Christians E (2010) Lack of maternal heat shock factor 1 results in multiple cellular and developmental defects, including mitochondrial damage and altered redox homeostasis, and leads to reduced survival of mammalian oocytes and embryos. Dev Biol 339, 338–353. 20045681 10.1016/j.ydbio.2009.12.037

[42] Ma X , Xu L , Alberobello AT , Gavrilova O , Bagattin A , Skarulis M , Liu J , Finkel T and Mueller E (2015) Celastrol protects against obesity and metabolic dysfunction through activation of a HSF1‐PGC1α transcriptional axis. Cell Metab 22, 695–708. 26344102 10.1016/j.cmet.2015.08.005

[43] Intihar TA , Martinez EA and Gomez‐Pastor R (2019) Mitochondrial dysfunction in Huntington's disease; interplay between HSF1, p53 and PGC‐1α transcription factors. Front Cell Neurosci 13, 103. 30941017 10.3389/fncel.2019.00103 PMC6433789

