# Supplementary material for: HSF1 is required for induction of mitochondrial chaperones during the mitochondrial unfolded protein response
Source: FEBS Open Bio. 2020 May 15;10(6):1135–48. doi: 10.1002/2211-5463.12863 (PMC7262932; doi:10.1002/2211-5463.12863)
Supplement: Supplementary file 1 — Table S1. Primer sequences used for RT‐qPCR. Table S2. Nucleotide sequences of shRNAs used for gene knockdown. Table S3. Primer sequences used for ChIP assay. [file FEB4-10-1135-s001.pdf]

Table S1. Primer sequences used for RT-qPCR

qPCR	Forward primer	Reverse primer
HSP70	5'-GGCTGGTGAGCCACTTCGT-3'	5'-GTTCTGGCTGATGTCCTTCTTGT-3'
mtHSP70	5'-AACGGCAAGCTGTCACCAA -3'	5'-ATCGTCGTCCAATAAGACGCTTA-3'
HSP60	5'-GGCACTGGCTCCTCATCTCA-3'	5'-GCGTCCGCACCAAATTTT-3'
HSP10	5'-TGCTGCCGAAACTGTAACCA-3'	5'-TGCAACACTTTTCCTTGAGACTTT-3'
LON	5'-TCCGACTTGCACAGCCCTAT-3'	5'-CGTCCGACTCATTGTTGTCATC-3'
GAPDH	5'-CGACTTCAACAGCAACTCCCACTCTTCC-3'	5'-TGGGTGGTCCAGGGTTTCTTACTCCTT-3'
RNPL0	5'-GAGGACCTCACTGAGATTCGG-3'	5'-TTCTGAGCTGGCACAGTGAC-3'

Table S2. Nucleotide sequences of shRNAs used for gene knockdown

shRNA	Sense strand	Antisense strand
SCR	5'-GATCCATGTACTGCGCGTGGAGACTTCAAGA GAGTCTCCACGCGCAGTACATTCTTTTGGAAA-3'	5'-AGCTTTTCCAAAAGAATGTACTGCGCGTGGA GACTCTCTTGAAATCAGTCGTATTTCTCTTCG-3'
TRAP1	5'-GATCCGGTTCTGGAGTGTTTGAAATCAAGAGA TTTCAAACACTCCAGAACCATTTTTTTGGAAA-3'	5'-AGCTTTTCCAAAAAAATGGTTCTGGAGTGTTTGA AATCTCTTGAATTTCAAACACTCCAGAACCG-3'
HSF1	5'-GATCCGTGATCACCTGGATGCCATTTCAAGA GAATGGCATCCAGGTGATCACTTTTTTGGAAA-3'	5'-AGCTTTTCCAAAAAAGTGATCACCTGGATGCCA TTCTCTTGAA ATGGCATCCAGGTGATCACG-3'
SSBP1	5'-GATCCAGTTTGGTTCTTGAACGATTTCAAGA GAATCGTTCAAGAACCAAACTGCTTTTGGAAA-3'	5'AGCTTTTCCAAAAGCAGTTTGGTTCTTGAACGATT CTCTTGAAATCGTTCAAGAACCAAACTG -3'

Table S3. Primer sequences used for ChIP assay

CHIP-qPCR	Forward sequence	Reverse sequence
HSP60/HSP10 promoter	5'-GCCGAGGTGAAAGAACGA-3'	5'-TCCCGTGGGTGAAAGGT-3'
mtHSP70 promoter	5'-ATGAGAGAACGGGAACTTTC-3'	5'-GAGACTACGTGTCCGCTGCC-3
HSP70.3 promoter (dHSE)	5'-ACCCTCCCCCTCAGGAATC-3'	5'TGTCCAGAACTCTCCAGAGGTTT-3'
HSP70.3 promoter (intergenic region)	5'-GTGGCGCATGCCTTTGAT-3'	5'- CTTTGTAGAACAGGCTGACCTTGA -3'
